# Pilot Evaluation of the Impact of Lottery-Based Incentives on Engagement Levels of Male Low SES Vocational Students With an mHealth App

**DOI:** 10.3389/fdgth.2021.748588

**Published:** 2022-01-07

**Authors:** Raoul Nuijten, Pieter Van Gorp, Juup Hietbrink, Pascale Le Blanc, Astrid Kemperman, Pauline van den Berg, Monique Simons

**Affiliations:** ^1^School of Industrial Engineering, Eindhoven University of Technology, Eindhoven, Netherlands; ^2^Department of the Built Environment, Eindhoven University of Technology, Eindhoven, Netherlands; ^3^Consumption and Healthy Lifestyles Group, Wageningen University & Research, Wageningen, Netherlands

**Keywords:** lottery-based incentive, regret lottery, anticipated regret, low SES, vocational students, health promotion, physical activity, dietary intake

## Abstract

In general, individuals with lower socioeconomic status (SES) are less physically active and adhere to poorer diets than higher SES individuals. To promote healthier lifestyles in lower SES populations, we hosted a digital health promotion program among male vocational students at a school in The Netherlands. In a pilot study, we evaluated whether this target audience could be engaged with an mHealth app using lottery-based incentives that trigger feelings of anticipated regret. Especially, we studied the social and interpersonal aspects of regret lotteries in a within-subject experimental design. In this design, subjects either participated in a social variant (i.e., with students competing against their peers for a chance at a regret lottery), or an individual variant (i.e., with subjects solely individually engaged in a lottery). Additionally, we studied the impact of different payout schedules in a between-subject experimental design. In this design, participants were assigned to either a short-term, low-value payout schedule, or a long-term, high-value payout schedule. From a population of 72 male students, only half voluntarily participated in our 10-week program. From interviews, we learned that the main reason for neglecting the program was not related to the lottery-based incentives, nor to the prizes that were awarded. Instead, non-enrolled subjects did not join the program, because their peers were not joining. Paradoxically, it was suggested that students withheld their active participation until a larger portion of the sample was actively participating. From the subjects that enrolled in the program (*N* = 36, males, between 15 and 25 years of age), we found that a large proportion stopped interacting with the program over time (e.g., after roughly 4 weeks). Our results also indicated that students performed significantly more health-related activities when assigned to the social regret lottery, as opposed to the individual variant. This result was supported by interview responses from active participants: They mainly participated to compete against their peers, and not so much for the prizes. Hence, from this study, we obtained initial evidence on the impact of social and competitive aspects in lottery-based incentives to stimulate engagement levels in lower SES students with an mHealth app.

## 1. Introduction

Over the past decade, public health literature has clearly demonstrated a relationship between socioeconomic status [SES, an individual's standing in the economic and social stratification system (1)] and unhealthy lifestyle behavior (1–3). In particular, it has been shown that lower SES individuals are generally less physically active and adhere to poorer diets than people with a higher SES (1). In order to support lower SES individuals to adopt healthier lifestyles, lifestyle interventions may be employed (4). Unfortunately, deploying lifestyle interventions among lower SES individuals remains challenging, as researchers in the past have struggled to access, and engage participants from lower SES populations (5, 6). As a result, lower SES groups are not well-represented in the populations that are typically studied in health promotion (5, 7). Vocational educational environments provide a potential setting to reach lower SES individuals, since socioeconomic status heavily correlates with educational level (1, 8). An added benefit of targeting vocational students (i.e., individuals in their (pre)adolescence) is that newly adopted lifestyle behaviors may potentially track into adulthood, which also ensures health benefits at a later stage in their lives (9, 10). Meanwhile, an educational environment provides a relatively safe setting in which students are likely to be willing to participate in pilot health promotion programs (11). Hence, this study was hosted within a vocational educational setting.

A challenge of lifestyle intervention design is to select the appropriate strategies to stimulate end user engagement with the intervention (4, 12). Gamification strategies are a promising set of motivational techniques that employ game mechanics outside game contexts in order to foster participation, engagement, and loyalty (13, 14). Gamification has often been hypothesized to be especially powerful at engaging lower SES individuals in lifestyle interventions, but the impact of gamification on this population has only been scarcely evaluated (7)]. From the set of gamification techniques (15), particularly the application of lottery-based incentives seems a promising strategy to engage our target group. Especially, because from both empirical data and survey-based studies it was consistently found that lower SES individuals engage in higher rates of lottery gambling than higher SES individuals (16). Moreover, a study by Haisley and colleagues evaluated the use of lottery-based incentives for the completion of health risk assessments and found that lower SES individuals had higher odds of completing the assessment than higher SES individuals when stimulated through a lottery-based incentive (17). Additionally, a study by Van der Swaluw and colleagues found that lottery-based incentives can increase physical activity levels and gym attendance in overweight adults (18). Particularly, in that study, regret lotteries were found to have a profound impact on participant engagement levels (18). In a regret lottery, prizes are drawn from all participants. Yet, non-eligible “winners” are not awarded; instead they are informed of their forgone prize (19). This type of incentive leverages anticipated regret–a negative emotion that we experience when realizing that our present situation would have been better, had we decided differently–and our tendency to avoid that emotion (19). This feeling likely triggers a participant to take action (e.g., by engaging in health-related activities), to minimize the chances of receiving a notification on a foregone prize. For example, Hussain and colleagues have demonstrated that the implementation of incentives that trigger feelings of anticipated regret can improve adherence with an intensive self-monitoring study protocol. They concluded that regret lotteries may represent a cost-effective tool to improve user engagement levels (20).

This study aims to extend the body of literature on the impact of regret lotteries on engagement levels with an mHealth app in lower SES populations. In this pilot study, we explored the impact on student engagement levels of either social, or individual regret lotteries, by controlling whether lottery outcomes were publicly announced (i.e., the social variant), or not (i.e., the individual variant). Because anticipated regret was proposed to be larger if also visible to (significant) others (19), we hypothesized that the impact of the social regret lottery-based incentive on engagement levels would be larger than the impact of the individual variant. Additionally, we explored the impact of varying payout schedules in terms of timing and monetary value. It was proposed that regret is larger for foregone prizes with a higher (monetary) value, and that regret is larger if the consequences start to materialize sooner (19). In a between-subject experimental design, we explored the trade-off between a short-term, low-value payout schedule and a long-term, high-value payout schedule, to explore which component (i.e., either the timing, or monetary value) of a reward has a more profound impact on end user engagement levels.

## 2. Materials and Methods

### 2.1. Recruitment

Participants were recruited among first-year and second-year (i.e., 15- to 20-year-olds), vocational students at a vocational school (i.e., MBO) in The Netherlands in April 2020. The entire population comprised 72 students, distributed over 6 different school classes. The study was advertised as a health promotion program that students joined voluntarily. The written consent of students was collected digitally upon registration for the program. All (operational) procedures were approved by the ethics committee of Eindhoven University of Technology (protocol code ERB2020IEIS22, approved May 7, 2020).

### 2.2. Intervention Context

For this study, we have developed a custom web interface using the mHealth app GameBus (21, 22), to suggest a set of health-related activities to participants [i.e., see (23) for detailed visualizations of the mHealth app]. The app awarded students with virtual points for performing these activities, based on a photo or video of the student performing the activity, to prove that the student engaged in that activity. Additionally, the app provided a newsfeed, which showed an entry for each activity from any student that was rewarded with points. Such entries could be liked or commented upon in a manner similar to mainstream social media platforms such as Facebook and Instagram. Also, the app provided a chat environment, where students could exchange messages with their own school class, or entire school. Moreover, the app included the lottery-based incentives custom to this study (i.e., details can be read in the next section). Prior studies that have evaluated the impact of lottery-based incentives have hosted and communicated their incentives outside the digital tool that supported their intervention (e.g., by sharing lottery outcomes via e-mail, or text messages) (18, 20). To provide a more integrated experience, we hosted the lottery-based incentives inside the our app (i.e., as is common in present-day entertainment games).

The overall goal of the intervention from the students' perspective was to obtain as many virtual points as possible by performing the suggested health-related activities. In particular, it was set by the school's management to focus on: (1) increasing physical activity, (2) improving dietary intake, and (3) fostering sustainable relationships among students [i.e., in line with contemporary conceptualizations of health, such as the Positive Health Philosophy (24)]. From these focal areas, a list of prescribed activities (i.e., including the number of points awarded per activity) was compiled. The aim was to define activities that students were capable of performing, that they would enjoy doing, and that would benefit their health. In a co-creation session (i.e., supervised by author RN), a group of two students and one teacher drafted a list of 43 unique activities that met these criteria. Eventually, the list included a broad range of activities (including e.g., “wrestle arms with someone of at least 40+,” or “make a healthy smoothie”). We have aimed at giving students ample opportunity for engaging in activities they would personally enjoy. Particularly, as it is known from motivational theories of (health) behavior change [e.g., the Self-Determination Theory (25)] that autonomous choice is essential to foster engagement levels.

The entire program lasted 10 weeks, and was divided into five program “waves,” each with a duration 2 weeks. Every wave, a subset of activities were suggested, see Supplementary Material for a complete overview of prescribed activities per wave and the number of virtual points awarded per activity. Eventually, all waves consisted of 11 unique activities, except for the third wave, which consisted of 12 unique activities. Each wave included a mix of activities from different focal areas. Some activities were duplicated over multiple waves, again see Supplementary Material for a detailed overview. Additionally, at the start of each wave, all participants received a notification via e-mail. The e-mail included instructions, for example on how to participate, or on the suggested activities to perform in a given wave. Furthermore, to inspire (passive) participants, the e-mail included a video compilation of randomly selected activities their peers had performed in the wave before. Finally, teachers were kindly requested to bring up the program in their classroom during teaching hours, such that students were regularly reminded of the program.

### 2.3. Study Design

To explore different aspects of meaningful (regret) lottery design in a health promotion context, we have designed an experiment with four study arms. In a within-subject experimental design, we have evaluated the impact of a social regret lottery-based incentive, compared to the impact of an individual lottery-based incentive. Subjects in both the first and third study arm (i.e., SA1 and SA3) were assigned to the individual lottery-based incentive in the first wave (i.e., and in the next waves alternately to the social regret lottery and the individual lottery, respectively). Subjects in both the second and fourth study arm (i.e., SA2 and SA4) were assigned to the social regret lottery-based incentive in the first wave (i.e., and in the next waves alternately to the individual lottery and the social regret lottery, respectively). Additionally, in a between-subject experimental design, we have evaluated the impact of a short-term, low-value payout schedule, compared to the impact of a long-term, high-value payout schedule. Subjects in SA1 and SA2 were assigned to the short-term, low-value payout schedule, whereas subjects in SA3 and SA4 were assigned to the long-term, high-value payout schedule. Lastly, participants were allocated to the different study arms using a randomized block approach (i.e., school classes were randomly distributed over the different study arms), resulting in a study design that was theoretically balanced with 14–20 students per study arm. The entire study design is detailed in Table 1. The next subsections provide more details on the actual implementations of the different lottery-based incentives.

**Table 1:** Overview of study arms and treatment allocation per school class.

**Study arm**	**School class**	**Between-subject**	**Within-subject*^a^***				
			**W1**	**W2**	**W3**	**W4**	**W5**
SA1	1 class, 14 students	Short-term, low-value	I	S	I	S	I
SA2	2 classes, 20 students		S	I	S	I	S
SA3	1 class, 19 students	Long-term, high-value	I	S	I	S	I
SA4	2 classes, 19 students		S	I	S	I	S

a * W, wave number; S, social regret lottery-based incentive; I, individual lottery-based incentive*.

#### 2.3.1. Social Aspects of (Regret) Lottery-Based Incentives

##### 2.3.1.1. Implementation of a Social Regret Lottery-Based Incentive

**Figure 1:**
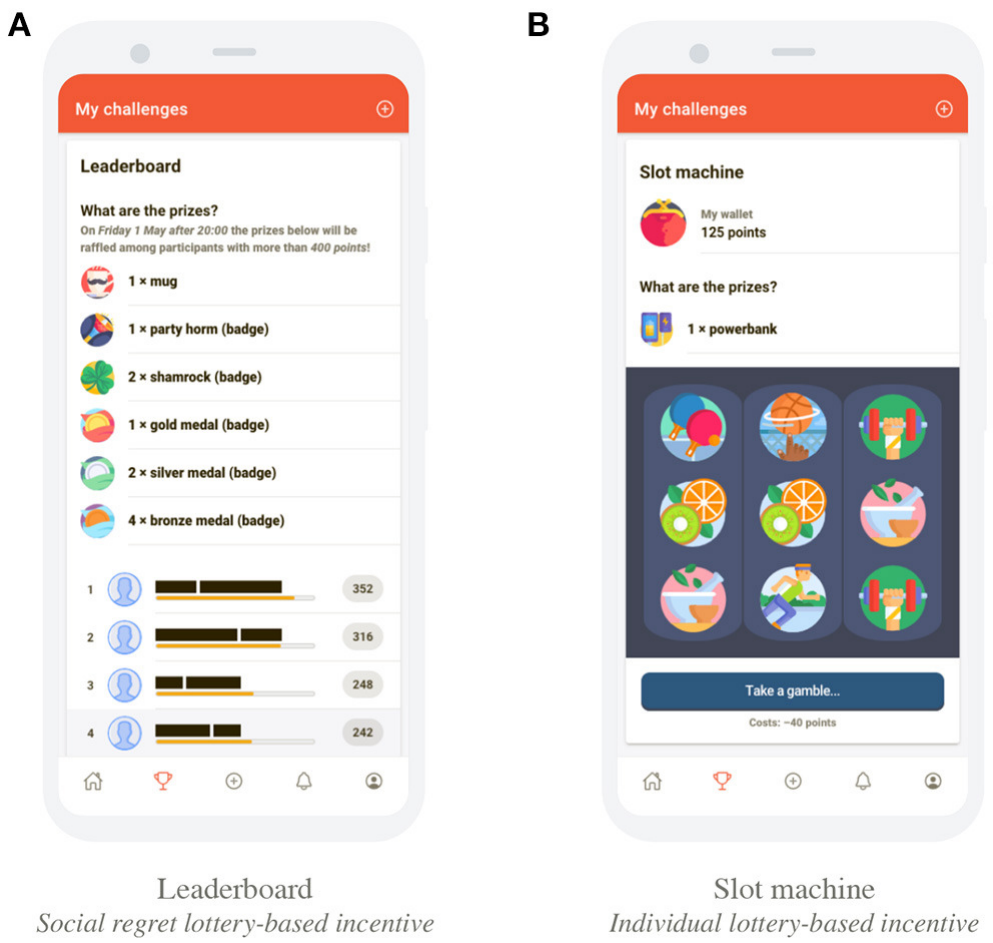
Display of the social regret lottery-based incentive and individual lottery-based incentive. See (23) for additional visualizations of the mHealth app. **(A)** leaderboard as the social regret lottery-based incentive, and **(B)** slot machine as the individual lottery-based incentive.

The social lottery-based incentive was visualized as a leaderboard, that displayed the total number of points a participant had obtained in a given wave, also see Figure 1A. At the end of a wave, prizes (i.e., both tangible rewards and virtual badges) were randomly distributed over participants with a number of virtual points greater than a target value. The target value to be eligible to win prizes was 400 points in the first wave, 100 virtual points in the second wave, and 50 virtual points in the last three waves. Winners were announced at the end of each wave via an in-app chat message that was visible to all participants within a particular study arm. Note that, to maximize anticipated regret, prizes were drawn from *all* participants, although non-eligible “winners” were not awarded, but instead they were publicly informed on their forgone prize. Also, participants could review each other's position on the leaderboard, and eligibility to win prizes at any time.

##### 2.3.1.2. Implementation of an Individual Lottery-Based Incentive

The individual lottery-based incentive was visualized as a digital slot machine, also see Figure 1B. Participants could exchange their virtual points for a spin of the slot machine. The costs of spinning were 40 virtual points in the first wave, 10 virtual points in the second wave, and 5 virtual points in the last three waves. Note that anticipated regret was minimal, as it was unclear if different actions (i.e., performing more activities, and spinning the slot machine more often) would have actually resulted in a better outcome (i.e., winning more prizes). Additionally, others were not informed on an individual's foregone prizes, thereby again minimizing anticipated regret.

#### 2.3.2. Aspects of Payout Schedules

The school's management had provided several tangible rewards that were likely attractive for the students. The school provided 54 tangible rewards. Hence, only roughly 11 tangible rewards were available per wave, which had to be distributed over a sample that could theoretically contain up to 72 students. To increase participant's overall chances to win a prize, several virtual badges were introduced. Note that these badges did not have any monetary value. The prizes (i.e., both tangible rewards and virtual badges) were distributed throughout the program in a between-subject experimental design, to explore the impact of varying payout schedules in terms of timing and monetary value.

##### 2.3.2.1. Implementation of a Short-Term, Low-Value Payout Schedule

Several prizes were distributed in a short-term, low-value payout schedule. The payout schedule was short-term as prizes were distributed regularly (i.e., at the end of every wave). The payout schedule was low-value because the prizes were of lower monetary value (i.e., between €2.50 and €15.00, also see Table 2 for an overview of rewards distributed per wave with the odds of winning prizes in the individual lottery-based incentives displayed in footnotes). Note that subjects could also win different virtual badges during the waves, though these badges did not have any monetary value. Note that in this scenario, anticipated regret was particularly triggered from the timing of the payout: Since payouts of rewards were scheduled every wave, consequences of actions materialized sooner, thereby potentially increasing anticipated regret.

**Table 2:** Overview of rewards per wave for the short-term, low-value payout schedule.

**Prize**	**Value**	**SA1**					**SA2**				
		**W1**	**W2**	**W3**	**W4**	**W5**	**W1**	**W2**	**W3**	**W4**	**W5**
Charging cable	€ 15.00		1				1				1
Power bank	€ 10.00	1*^a^*				1*^d^*		1*^a^*			
Multi-tool	€ 2.50			1*^a^*						1*^a^*	
Notebook	€ 2.50				1				1		1
Bike gadget	€ 2.50										1
Smartphone holder	€ 5.00					1*^d^*					
Mug	€ 7.50	2*^b^*	2	2*^b^*	2	2*^d^*	2	2*^b^*	2	2*^b^*	2
Party horn badge	€ 0		1		1		1		1		1
Shamrock badge	€ 0		2		2		2		2		2
Jackpot badge	€ 0	1*^a^*		1*^a^*		1*^d^*		1*^a^*		1*^a^*	
Lucky bird badge	€ 0	2*^b^*		2*^b^*		2*^d^*		2*^b^*		2*^b^*	
Gold badge	€ 0	1*^b^*	1	1*^b^*	1		1	1*^b^*	1	1*^b^*	
Silver badge	€ 0	2*^c^*	2	2*^c^*	2		2	2*^c^*	2	2*^c^*	
Bronze badge	€ 0	4*^d^*	4	4*^d^*	4		4	4*^d^*	4	4*^d^*	
**Total value**		€120.00					€125.00				

a *odd = 0.01*;

b *odd = 0.05*;

c *odd = 0.10*;

d *odd = 0.15*.

##### 2.3.2.2. Implementation of a Long-Term, High-Value Payout Schedule

Several prizes were distributed in a long-term, high-value payout schedule. The payout schedule was long-term as prizes were distributed at the end of the program. The payout schedule was high-value because the prizes were of higher monetary value (i.e., up to €100.00), see Table 3 for more details on the different rewards that were available. Again, the odds of winning prizes in the individual lottery-based incentive are displayed in footnotes. Note that subjects could also win different virtual badges during the waves, though these badges did not have any monetary value. The schedule was long term, because the grand prize (i.e., a hard drive worth €100.00) was only be distributed at the end of the program. Particularly, the grand prize was raffled over participants in both SA3 and SA4 with at least 400 virtual points at the end of the program. Lastly, note that anticipated regret was in this scenario particularly triggered from the value of the payout. Since payout of monetary rewards were scheduled at the end of the program, consequences of actions materialized later, thereby lowering anticipated regret. At the same time, the monetary value of rewards was significantly larger, thereby potentially increasing anticipated regret.

**Table 3:** Overview of rewards per wave for the long-term, high-value payout schedule.

**Prize**	**Value**	**SA3**					**SA4**				
		**W1**	**W2**	**W3**	**W4**	**W5**	**W1**	**W2**	**W3**	**W4**	**W5**
Hard drive	€ 100,00	1									
Power bank	€ 10.00										1
Smartphone holder	€ 5.00					1*^e^*					
Multi-tool	€ 2.50					1*^e^*					1
Bike gadget	€ 2.50										1
Mug	€ 7,50	1*^c^*	2	1*^c^*	2	1*^e^*	2	1*^c^*	2	1*^c^*	2
Party horn badge	€ 0		1		1		1		1		1
Shamrock badge	€ 0		2		2		2		2		2
Jackpot badge	€ 0	1*^a^*		1*^a^*		1*^e^*		1*^a^*		1*^a^*	
Lucky bird badge	€ 0	2*^b^*		2*^b^*		2*^e^*		2*^b^*		2*^b^*	
Gold badge	€ 0	1*^b^*	1	1*^b^*	1		1	1*^b^*	1	1*^b^*	
Silver badge	€ 0	2*^d^*	2	2*^d^*	2		2	2*^d^*	2	2*^d^*	
Bronze badge	€ 0	4*^e^*	4	4*^e^*	4		4	4*^e^*	4	4*^e^*	
**Total value**		€110.00					€125.00				

a *odd = 0.01*;

b *odd = 0.05*;

c *odd = 0.06*;

d *odd = 0.10*;

e *odd = 0.15*.

### 2.4. Measurements

#### 2.4.1. Objective Measures of Engagement

In mHealth research, engagement is most commonly captured via measures of app usage (26, 27). Using the GameBus platform, engagement of participants has been repeatedly measured as: (1) the number of days a participant had been online (i.e., distinct days the participant opened the app), and (2) the number of activities a respondent performed. These variables complement each other since the former may be limited to passive engagement, while the latter requires active participation (i.e., performing the suggested activities). Both measurements were recorded per participant per wave. For each record, also the wave number relative to the respondent's participation date was recorded. Hence, a record for a particular subject who joined the program only in the fifth wave, would have a relative wave number of one for that record. Finally, users were categorized in one of three categories, depending on their actual engagement level. Subjects that did not enroll in the program were labeled as *non-enrolled users*. Students that enrolled in the program, but did not engage in the suggested activities were labeled *passive users*. Finally, subjects that enrolled in the program and did register at least one health-related activity were labeled *active users*.

#### 2.4.2. Subjective Measures of Engagement

A post-test survey was used to collect demographic data (e.g., age and gender) from the entire target population. The survey was distributed at the end of the program to the entire target population. Students were allowed to complete the post-test survey during classroom hours. Additionally, at the end of the program, two semi-structured, focus group interviews were conducted to further elaborate on participants motivation to either participate, or not to participate. The first focus group panel consisted of three randomly selected participants that did not register any activities into the mHealth app, whereas the second focus group panel consisted of three randomly selected participants that were among the participants with the highest number of activity registrations. The interviews were conducted online, via videoconferencing software, and were supervised by two authors (i.e., JH and RN). The main focus of the interviews has been to qualitatively derive: (1) the students' preferences for either the social variant, or individual variant, of the lottery-based incentives, and (2) the students' perceptions of attractiveness of the tangible rewards. Additionally, within the panel of non-enrolled participants, we have also focused at deriving the barriers that withheld them from participating in the program. Finally, within the panel of active participants, we have also aimed to derive the students' perceptions of the impact of the program on their overall health status and engagement in health behaviors.

### 2.5. Statistical Analysis

Our statistical analyses were focused at evaluating the engagement levels of respondents. For both outcome variables (i.e., the number of days a participant had been online, and the number of activities a respondent had performed), exploratory analyses were performed using mean plots to detect potential differences between study arms, over time. Subsequently, several generalized (i.e., Poisson) hierarchical linear models were estimated for these two outcome variables, using time (i.e., the relative wave number), and the treatment variables (i.e., the incentive type–either social, or individual–and the payout schedule–either short-term, low-value, or long-term, high-value) as predictors. To obtain the final models for both outcome variables, a backward elimination selection procedure was employed (28). Backward elimination starts with all predictors (i.e., the relative wave number, the incentive type, and payout schedule) included in the model, with variables subsequently being eliminated, one at the time. At each step, the predictor with the highest *p*-value with *p*>0.05 is deleted (28). This method of deletion continues until all predictors are significant (i.e., *p* ≤ 0.05). We have not tested whether significant second-order interaction effects existed amongst these variables, as sample sizes in individual clusters would then have been too low for a thorough analysis of second-order interaction effects. In all models we did allow for random intercepts between individual participants. Subsequently, we have used Pearson's Chi-Squared test to confirm our final models do not suffer from overdispersion. Overdispersion of a Poisson model occurs when the variance value of the underlying data is greater than the mean value of that data, suggesting that the data is not likely to be modeled well using a Poisson model (29). Lastly, we have checked that our final models do not suffer from zero-inflation, too much. A zero-inflated Poisson model may predict an excess number of zero counts, that is not representative of the Poisson distribution anymore. In this study, the ratio between the predicted number of zero counts, and the actual number of zero counts is preferred to be greater than or equal to 0.95. Finally, based on the digital recordings of the two focus group interviews, we have selected a set of quotes that all participants within a focus group panel (i.e., three interviewees per panel) did explicitly agree to. The selection of quotes was performed by three authors (i.e., JH, PVG, and RN).

## 3. Results

### 3.1. User Statistics

**Figure 2:**
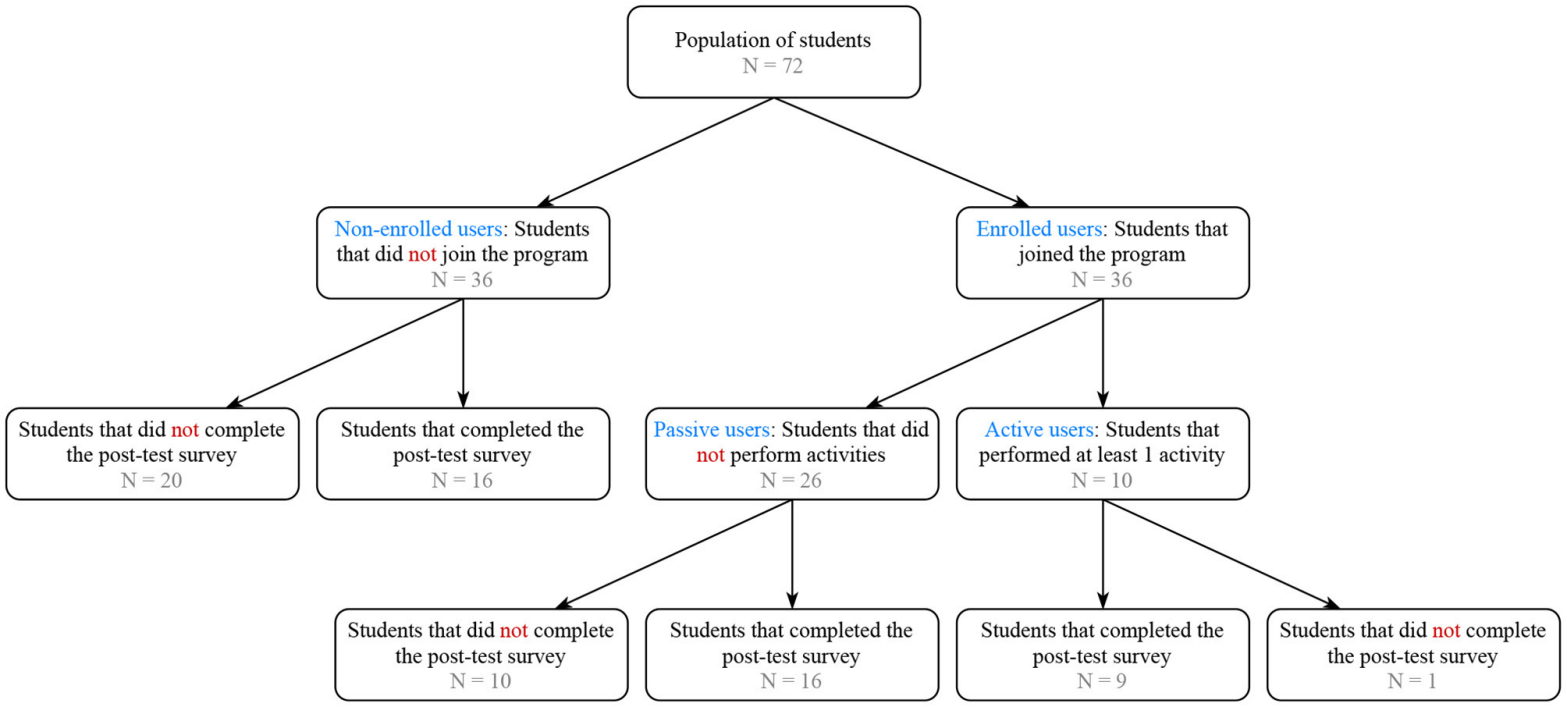
Cohort diagram that details the number of students enrolled in different study phases.

In total, 38 unique participants joined the study. Amongst the participants were 36 students, and 2 teachers (but teachers were excluded in further statistical analyses). The students were assigned to the different study arms using a randomized block approach: 6 students were assigned to SA1, 17 to SA2, 2 to SA3, and 11 to SA4. Of the 36 students enrolled, 10 students performed at least one activity; the others have only been checking the app. Figure 2 displays a cohort diagram which details the number of students enrolled in different study phases. The post-test survey was completed by 41 students, of which 25 students were enrolled in the program (i.e., they created a user account), also see Figure 2. The entire population was reported to be male, 28 students were 18+, and 13 students were less than 18 years of age (i.e., based on 41/72 subjects that completed the post-test). Within our sample, 15 students were 18+, and 10 students were less than 18 years of age (i.e., based on 25/36 participants that enrolled in the program and completed the post-test). A total of 20 students have visited our app in the first wave, 22 in the second wave, 7 in the third wave, and 8 individuals visited our app in fourth and fifth waves.

### 3.2. Evaluation Outcomes

#### 3.2.1. Impact on Average Number of Days Online

**Figure 3:**
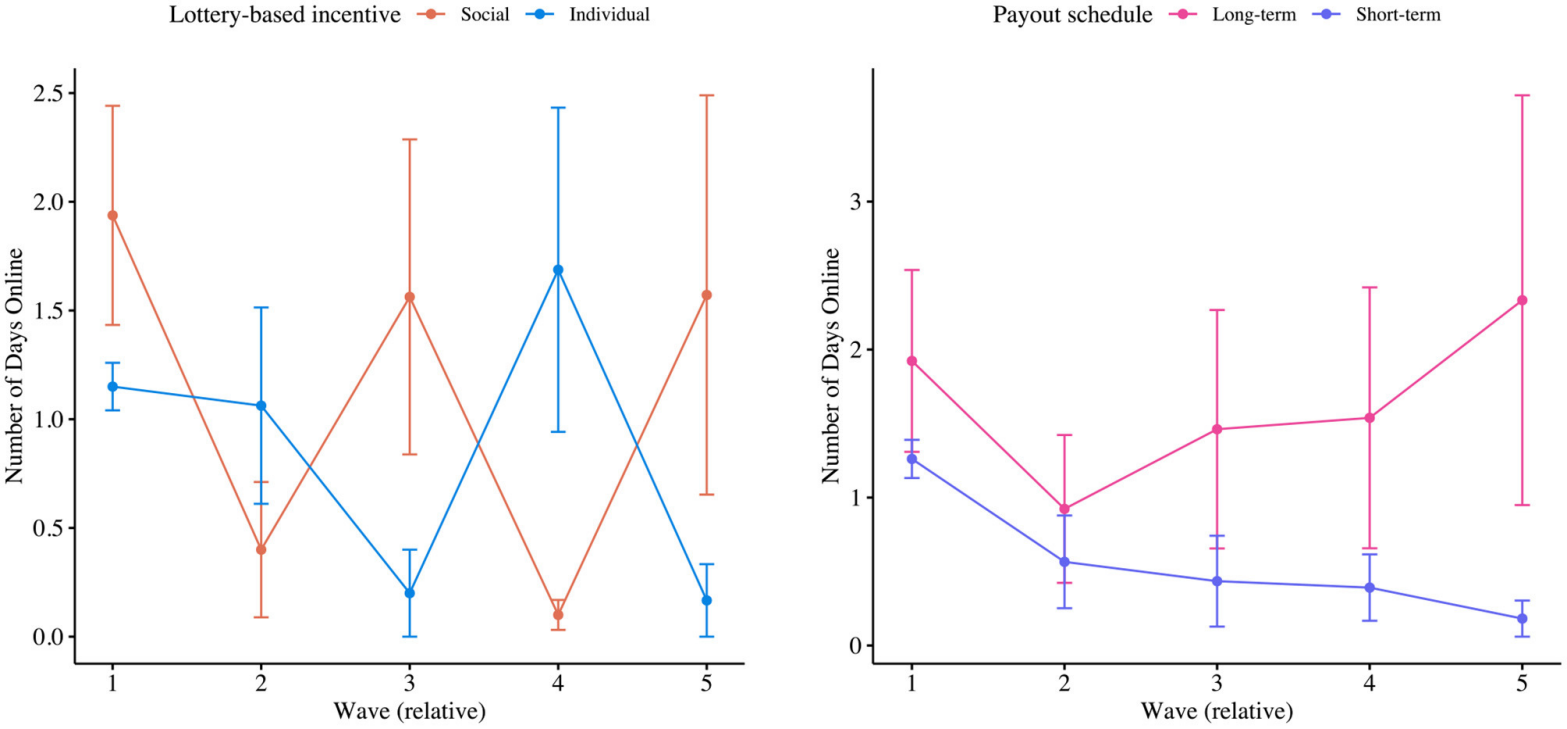
Mean plots of the number of days participant checked the app per type of incentive and per payout schedule, respectively.

Passive engagement was measured as the number of days participants checked the app within a given wave. Figure 3 displays the number of days participants were online (i.e., checked the mHealth app) on average per wave, both per type of incentive and payout schedule. Note that the sloped horizontal lines in these mean plots are a visual aid to highlight differences between (treatment) group averages.

From a backward elimination selection procedure we have obtained a final model for predicting the number of days participants checked the app with just one significant predictor. Particularly, we found that the number of days participants checked the app significantly decreased over time (i.e., −0.146 days per wave, at *p* = 0.01). The model was validated not to be harmed from overdispersion. Still, the model may have underfitted zeros, slightly (i.e., zero-inflation ratio was 0.94, instead of ≤ 0.95). Finally, although payout schedules were not reported to have a significant impact on passive levels of engagement, Figure 3 suggests that the long-term, high-value payout schedule especially fostered passive engagement levels in the fifth wave. As outlined before, we have not tested whether significant second-order interaction effects existed amongst, for example, the relative wave number and payout schedule, as sample sizes in individual clusters would then have been too low for a thorough analysis of second-order interaction effects (i.e., only a total of 8 students engaged with our app in the last wave). Still, Figure 3 may indicate that long-term, high-value payout schedules particularly foster passive engagement levels as its deadline approaches.

#### 3.2.2. Impact on Average Number of Activities

**Figure 4:**
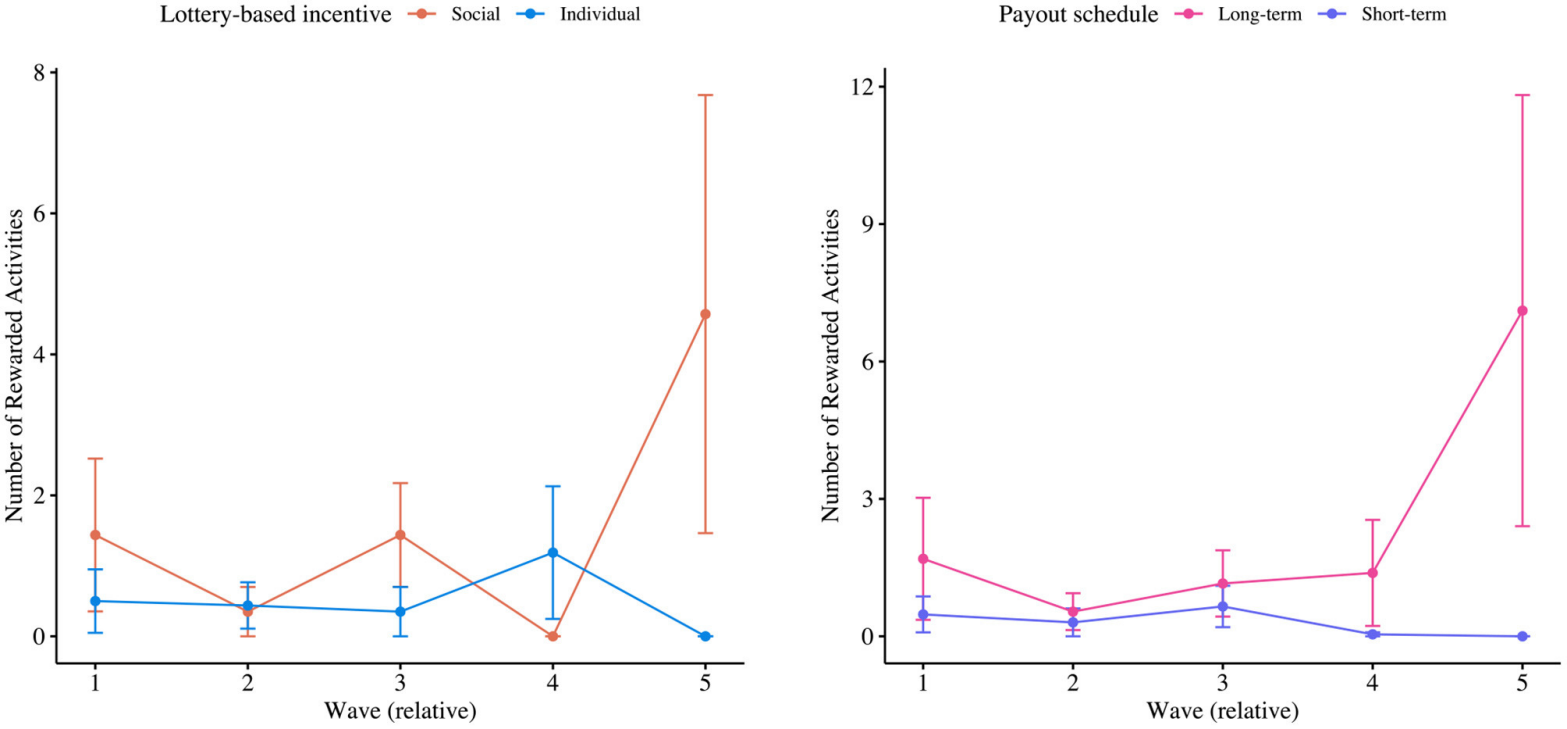
Mean plots of the number of activities participants registered per type of incentive and per payout schedule, respectively.

Figure 4 displays the average number of activities participants have performed per wave, both per type of incentive and payout schedule. From an additional backward elimination selection procedure to derive a model for predicting the number of activities participants performed, we have obtained a final model with two predictors. Particularly, we found that the number of activities participants performed significantly increased over time (i.e., +0.209 activities per wave, at *p* < 0.001). Additionally, it was found that the number of activities participants performed decreased when engaged with the individual lottery-based incentive (i.e., −0.726 activities, at *p* < 0.001), as opposed to the social lottery-based incentive. The model was validated not to be harmed from overdispersion and not to be underfitting zeros (i.e., with a zero-inflation ratio of 0.95). Finally, again, Figure 4 seems to suggest that the long-term, high-value payout schedule especially fostered active engagement levels in the fifth wave. Again, we have not tested whether significant second-order interaction effects existed amongst (i.e., because only a total of 8 students engaged with our app in the last wave). Still, Figure 4 may again indicate that long-term, high-value payout schedules particularly foster active engagement levels as its deadline approaches.

### 3.3. Focus Group Interview Evaluation

#### 3.3.1. Focus Group Interview With Non-enrolled Participants

As the main reason for not engaging with the program, participants within the panel of non-enrolled participants designated the overall low uptake with the program: “*I only would have participated in the program when more people would have joined*.” Additionally, a participant mentioned: “*I would have liked to engage in a competition with my peers, but when I checked the leaderboard, I did not see too many active participants, which in turn did not spark my interest to participate*.” Another reason for not engaging with the program was that interviewees found the suggested activities and prizes insufficiently attractive: “*Although the suggested activities and prizes looked like they could have been attractive for some, they did not stimulate me to engage with the program*.” Nevertheless, the interviewees even speculated that prizes with a higher monetary value would also not have triggered them to actively participate: rather would they have engaged in a program with a higher overall uptake. Lastly, interviewees were hesitant to participate because of (potential) invasions of privacy: “*I did not like the fact that I was required to upload photos and videos of myself engaging in the suggested activities, as I felt my privacy could thereby potentially be invaded*.”

#### 3.3.2. Focus Group Interview With Active Participants

Participants within the panel of active participants agreed that they “*did not participate to win prizes*“: their main incentive was “*to engage in a competition with other participants*.” One focus group interviewee mentioned that he “*wanted to be better than the others*.” He added: “*Especially, I wanted to beat the ones with a comparable number of virtual points as myself: even when we could not see from each other the number of points we had collected [because we were engaged in the individual lottery-based incentive], we kept each other posted on the number of points we had collected*.” “ *Whenever I was falling behind, I would make sure to perform more activities to catch up*.” The drive to compete against others made participants hesitant to exchange their virtual points for a spin of the slot machine in the individual lottery-based incentive. One subject mentioned that he did not want to spend his points at the digital slot machine, because he was “*afraid to loose his points*.” He mentioned: “*I was told that an other participant had spent all his points without winning anything, so I rather kept my points*.” Lastly, participants were unsure whether the program empowered them to change their lifestyle: “*I cannot say that the [mHealth] app changed my behavior, because I already engaged in physical activities regularly before the intervention*.” However, he mentioned: ‘*The [mHealth] app made engaging in healthy lifestyle behaviors more fun, especially when we could compete against each others by collecting points*.” Participants agreed that the implementation of (push) notifications could further increase the impact of the intervention.

## 4. Discussion

### 4.1. Principal Findings

In this pilot study we evaluated engagement levels of vocational students with a health promotion program that employed lottery-based incentives. Half of the target population of 72 male students voluntarily engaged with the program and were exposed to the lottery-based incentives. The other half of the population had never engaged with the program. From focus group interviews, we learned that the main reason for non-enrolled subjects to neglect the program was not related to the lottery-based incentives, nor the prizes that were awarded. Instead, non-enrolled subjects did not join the program since their peers were not joining the program. In other words, we found that the program would have been more engaging if more students had actively participated initially. This seems to be a paradox of participation: subjects withhold their active participation until a larger portion of the sample is actively participating. However, if everyone is waiting for their peers to participate, no one will participate. This paradox may potentially be resolved by introducing interdependencies among participants (22), such that individuals require the active participation of others in order to progress themselves. In the current implementation, it was actually beneficial to be the only active participant in both lottery-based incentives, as this would increase one's chances of winning (tangible) rewards.

From our analyses of subjects that did actually enroll in the program, we found that a large proportion of participants stopped interacting with the program over time (e.g., after participating for 2 waves, with a total duration of 4 weeks). A reason may be that students tried to minimize potential feelings of regret by ignoring the study (30). Another reason may be that students forgot about the program, or found the program insufficiently engaging. Nevertheless, although results have to be carefully interpreted due to relatively low sample sizes, from the analyses of engagement levels it was found that the social regret lottery-based incentive stimulated active user engagement, as participants performed significantly more activities when assigned to this variant (i.e., compared to the individual variant). Despite relatively large levels of variance within our sample, it was also reported in the focus group interviews with active participants that they found the social regret lottery-based incentive to be more stimulating.

Finally, from our exploratory statistical analyses of user engagement levels it was suggested that the long-term, high-value payout schedule fostered student engagement levels, especially in the last wave of the program. Although we could not statistically validate this observation (i.e., as sample sizes in individual clusters would have been too low for a thorough analysis of second-order interaction effects), this observation may indicate that the impact of a long-term, high-value payout schedule is largest when the deadline approaches (i.e., when long-term becomes short-term). Hence, in line with the proposition of Zeelenberg (19), a short-term, low-value payout schedule may also be employed to foster engagement, particularly at the beginning of a program, if the prize is sufficiently large. Note however, that from the focus group interviews we learned that students found the prizes attractive, but that they mainly participated to compete against their peers, indicating that the incorporation of social aspects in lottery-based incentives potentially has a larger effect on user engagement levels.

### 4.2. Limitations

This study was subject to several limitations. First, this study evaluated the impact of our intervention on a particular target group (i.e., vocational students) within a specific context (i.e., the school environment). Based on the current pilot study, findings cannot be generalized yet as results were derived from a small target population (i.e., *N* = 72) that only included male subjects. Moreover, only 36 out of 72 students enrolled in the study and, through our randomized block design, subjects were unevenly distributed to study arms (i.e., 6 subjects in SA1, 17 subjects in SA2, 2 subjects in SA3, and 11 subjects in SA4). Although measures were taken to attract as many students as possible to join the program (e.g., via repeated teacher invitations), as well as to balance sample sizes over study arms (e.g., in theory each study arm should have included 14 to 20 subjects), students participated in the program voluntarily. As a result, the overall sample size and sample sizes over study arms could not be controlled.

Second, we have not employed a control group in our study design, that would allow us to compare our treatments to a situation without lottery-based incentives. The control condition was not included, because the study organizer (i.e., the vocational school) had expressed a desire for all participating students to have the opportunity to win a tangible reward.

Third, additional data measures could have helped to better interpret the results. In particular, we have not measured the levels of regret participants actually perceived from the different lottery-based incentives. Similarly, no data was recorded on the number of times teachers brought up the program in their virtual classrooms, nor the degree(s) of students attending to these classrooms, which could have helped to explain adoption rates. Besides, the study could have benefited from an exploration of social relationships between students in order to be able to assess which students were befriended, which students were most popular and which teachers were beloved. Potentially, this analysis could have helped to target the most influential subjects, and motivated them to actively participate. Presumably, these subjects could have then triggered others to actively participate [e.g., as close relationships among lower SES students have been found to influence participation rates, at least in physical activity participation (31)].

Fourth, the experimental setup was vulnerable for fraudulent usage. In theory, participants could upload photos and videos of activities that were not performed by themselves, or that they had already used to claim points. The research team has validated incoming activities on a day by day basis. When any form of fraud was detected in an activity registration, all the points and possible prizes that were obtained from that activity were withdrawn. Participants who committed fraud were also alerted by a pop-up that their user account could be suspended, whenever they continued cheating [i.e., see (23) for detailed visualizations of the fraud detection procedure]. Throughout the study, no accounts were suspended, but the points from 7 activities that were registered by 3 different students were withdrawn for not including a valid photo or video (i.e., a valid photo or video proved that the student had actually engaged in the activity).

Finally, since this study was executed in times of the COVID-19 pandemic, students were not actually physically present at school. Instead, students were educated via videoconferencing. Hence, also our intervention, including participant recruitment by teachers, was hosted entirely online. This may have harmed the effectiveness of the participant recruitment strategy.

### 4.3. Future Work

To counter the paradox of participation that we identified, and to persist with momentum throughout the program, follow-up studies may evaluate the impact of creating interdependencies between participants, as well as using bot accounts to artificially boost the number of active participants. Additionally, follow-up studies could explore combinations of different payout schedules to find the optimal configuration for sustaining user engagement over a longer period of time. Lastly, follow-up studies could benefit from recording participants' levels of perceived regret from different lottery-based incentives. These measurements provide insight into the degree of anticipated regret vocational students perceive from different implementations of lottery-based incentives.

## 5. Conclusions

We found that lottery-based incentives that trigger feelings of anticipated regret can potentially be employed in lifestyle interventions to promote engagement with mHealth apps among lower SES vocational students. However, besides these lottery-based incentives, practitioners may want to employ other methods to initially attract a larger portion of the population to enroll in the program. Sole deployment of lottery-based incentives seems insufficient to attract an entire target population. But, paradoxically, a higher uptake may increase the actual impact of the lottery-based incentive itself. Additionally, the impact of lottery-based incentives may be fostered by including social and interpersonal (e.g., competitive) aspects. Particularly, we found that an incentive with social and competitive elements was more engaging than an incentive solely at the individual level. Finally, we have found initial evidence of payout schedules increase engagement, particularly when their deadline approaches. Hence, different payout schedules with alternating deadlines may potentially be employed to foster engagement at different phases throughout a lifestyle intervention. Still this observation has to be studied in more depth.

## Data Availability Statement

The datasets generated for this study can be found in figshare at 10.6084/m9.figshare.14386754, (23).

## Ethics Statement

The studies involving human participants were reviewed and approved by Ethics Committee of Eindhoven University of Technology. The written consent of students was collected digitally upon registration for the program. Written informed consent from the participants' legal guardian/next of kin was not required to participate in this study in accordance with the national legislation and the institutional requirements. Written informed consent was not obtained from the minor(s)' legal guardian/next of kin for the publication of any potentially identifiable images or data included in this article.

## Author Contributions

RN and PV: conceptualization, methodology, software, and validation. RN and JH: formal analysis, investigation, resources, and writing—original draft preparation. RN: data curation and visualization. RN, PV, PL, AK, PB, and MS: writing—review and editing and supervision. RN, PV, and JH: project administration. PV, PB, AK, PB, and MS: funding acquisition. All authors have read and agreed to the published version of the manuscript.

## Funding

This work was part of the research program Gamification for Overweight Prevention and Active Lifestyle (443001101), which was partly financed by the Netherlands Organization for Health Research and Development (ZonMw).

## Conflict of Interest

The authors declare that the research was conducted in the absence of any commercial or financial relationships that could be construed as a potential conflict of interest.

## Publisher's Note

All claims expressed in this article are solely those of the authors and do not necessarily represent those of their affiliated organizations, or those of the publisher, the editors and the reviewers. Any product that may be evaluated in this article, or claim that may be made by its manufacturer, is not guaranteed or endorsed by the publisher.
