# Supplementary material for: Pilot Evaluation of the Impact of Lottery-Based Incentives on Engagement Levels of Male Low SES Vocational Students With an mHealth App
Source: Front Digit Health. 2022 Jan 7;3:748588. doi: 10.3389/fdgth.2021.748588 (PMC8782146; doi:10.3389/fdgth.2021.748588)
Supplement: Supplementary file 1 [file Data_Sheet_1.PDF]

Overview of prescribed activities per wave

Cat.[a]	Suggested activity	Points	Proof	Waves				
PA	Shoot a ball on the crossbar of a goal	25	Video	.	.	.	4	.
PA	Walk 3 kilometers with a dog	25	Video	.	.	.	.	5
PA	Complete a workout via YouTube	8	Video	.	.	.	.	5
PA	Do 100 push-ups	5	Video	.	.	3	.	.
PA	Go up and down the stairs 10 times	5	Video	1	.	.	.	.
PA	Do some gardening	5	Video	.	.	.	4	.
PA	Do 30 lunges	4	Video	.	.	.	4	.
PA	Do a sports game that has you moving	4	Video	.	2	.	.	.
PA	Do 30 squats	4	Video	1	.	.	4	.
PA	Jump rope for 1 minute	4	Video	.	.	3	.	.
PA	Do 10 burpees	4	Video	1	.	.	5	.
PA	Cover 2 kilometers in 10 minutes	4	Video	.	.	.	.	5
PA	Run to the supermarket	4	Video	.	.	3	.	.
PA	Do an activity that makes you sweat	4	Video	1	2	3	4	5
PA	Clean your room with a vacuum cleaner	4	Video	.	.	3	.	.
PA	Empty the dishwasher	4	Video	.	.	.	.	5
PA	Imitate this yoga pose: 🧘	4	Video	.	2	.	.	.
PA	Do 20 push-ups	3	Video	1	.	.	.	5
PA	Do 40 crunches	3	Video	.	2	.	.	.
PA	Find an object weighing exactly 15 kilos and place it on a scale	3	Video	.	2	.	.	.
PA	Go for a bike ride	3	Video	.	2	.	.	5
DI	Cook a meal with potatoes, meat & vegetables	5	Photo	1	.	.	.	.
DI	Take a picture of a shopping cart with ingredients for a healthy meal	5	Photo	.	2	.	.	.
DI	Take a picture of a healthy lunch	5	Photo	.	.	3	.	.
DI	Drink 1 liter of water throughout the day	4	Photo	.	.	.	4	.
DI	Make a healthy smoothie	4	Photo	.	2	.	.	.
DI	Fry an egg	3	Photo	1	.	.	.	.
DI	Peel a hard-boiled egg	3	Photo	.	.	.	.	5
DI	Bake a pancake	3	Photo	.	.	3	.	.
DI	Peel an apple peel of at least 20 centimeters	3	Photo	.	.	.	4	.
DI	Make an elephant from a tangerine	3	Photo	1	.	.	.	.
DI	Have a cup of tea	2	Photo	.	.	3	.	.
FR	Get to know yourself: complete a personality test (i.e., using the mini-IPIP scales **?**	25	Photo	.	.	3	.	.
FR	Wrestle arms with someone of at least 40+	25	Photo	.	.	3	.	5
FR	Watch a documentary on video game addiction	6	Photo	.	.	.	4	.
FR	Perform an act of kindness in times of the COVID-19 pandemic	5	Photo	1	.	.	.	.
FR	Play a game of chess	4	Photo	1	.	.	.	.
FR	Play a game of checkers	4	Photo	.	.	3	.	.
FR	Play a game of Ludo	4	Photo	.	.	.	4	.
FR	Play a game of Connect Four	4	Photo	.	.	.	.	5
FR	Play a game of cards	4	Photo	.	2	.	.	.
FR	Skype with friends while doing a healthy activity	4	Photo	1	2	3	4	5
FR	Make a person laugh	3	Photo	1	.	.	.	.

[a] PA = physical activity; DI = dietary intake; FR = friends and relationships
